# Oligofructose supplementation (10%) during pregnancy and lactation does not change the inflammatory effect of concurrent trans fatty acid ingestion on 21-day-old offspring

**DOI:** 10.1186/1476-511X-12-59

**Published:** 2013-05-01

**Authors:** Ana Claudia Losinskas Hachul, Laís Vales Mennitti, Juliana Lopes de Oliveira, Mayara Franzoi Moreno, Marcos Hiromu Okuda, Bruno dos Santos, Lila Missae Oyama, Eliane Beraldi Ribeiro, Claudia Maria Oller do Nascimento, Luciana Pellegrini Pisani

**Affiliations:** 1 Departamento Fisiologia, Disciplina de Fisiologia da Nutrição, Escola Paulista de Medicina, Universidade Federal de São Paulo, Rua Botucatu, 862, 2º andar, Vila Clementino, São Paulo, SP, Brazil; 2 Departamento de Biociências, Instituto de Saúde e Sociedade, Universidade Federal de São Paulo, Santos, SP, Brazil

**Keywords:** Hydrogenated fat, Oligofructose, White adipose tissue, Muscle, Pregnancy, Lactation, Cytokines

## Abstract

**Background:**

Previously, we demonstrated that trans fatty acid ingestion during pregnancy and lactation caused a pro-inflammatory effect on the newborn. The opposite effect was described for gestational prebiotic intake. In the present study, we examined whether supplementation of the diet of the dams with 10% of oligofructose with or without hydrogenated vegetable fat during pregnancy and lactation affected the pro-inflammatory status on the pups at age 21 days.

**Methods:**

On the first day of pregnancy, rats were divided into four groups, each of which received one of four diets: a control diet (C group), a control diet supplemented with 10% oligofructose (CF group), a diet enriched with hydrogenated vegetable fat containing trans fatty acids (T group) or a diet enriched with hydrogenated vegetable fat containing trans fatty acids supplemented with 10% oligofructose (TF group). The pups were weighed at birth and at 7, 14 and 21 days of life and were euthanized on post-natal day 21. The serum glucose, insulin and adiponectin concentrations were analyzed. The IL-6, IL-10 and TNF-α contents of the retroperitoneal white adipose tissue, liver, soleus and extensor digital longus muscles were analyzed by ELISA. The results are presented as the means ± standard error of the mean. Statistical significance was assessed using two-way ANOVA, followed by Tukey's test and considered significant at *p* < 0.05

**Results:**

The body weights of the 21-day old pups in the CF and TF groups were significant lower than those of the C (27% and 21%) and T (25% and 19%, respectively) groups. The serum levels of adiponectin in the CF, T and TF groups were lower than in the C group (41%; 34% and 31%, respectively). In the retroperitoneal adipose tissue, the IL-6 content was increased in TF group relative to the C and CF groups (74% for both), and the TNF-α content was higher in the T and TF groups than in the C group (62% and 98%, respectively). In the liver, the TNF-α (56% and 104%) and IL-10 (52% and 73%) contents were increased in the CF group relative to the C and TF groups.

**Conclusions:**

Supplementation of the diet of the dams with 10% of oligofructose during pregnancy and lactation, independent of supplementation with hydrogenated vegetable fat, adversely affected the development of the offspring and contributed to development of a pro-inflammatory status in the pups on postnatal day 21.

## Introduction

Maternal nutrition during the course of pregnancy and during lactation plays a critical role in the development of the fetus, newborn and future adult by epigenetic modifications resulting in a metabolic imprint that induces changes in phenotype. Recently, the concept of metabolic programming has been applied to possible beneficial or adverse influences of the maternal nutritional supply to the fetus and newborn [1].

During fetal development, inadequate nutrition may alter one or more aspects of physiological and morphological development, which may increase the predisposition of the individual for developing metabolic disorders [2-4]. Currently, it is known that there is a correlation between the production of adipokines by adipose tissue and development of metabolic disorders [5].

Intake of hydrogenated vegetable fat, rich in trans fatty acids (TFAs), during pregnancy and lactation caused changes in the metabolism and decreased serum adiponectin of the offspring assessed at 21 days after birth. These changes were accompanied by increases in gene expression of TNF-α and protein expression of TRAF-6, a cytoplasmic protein associated with the toll-like receptor 4 (TLR-4) pathway [6,7], in adipose tissue. It has also been demonstrated that the consumption of TFAs by rodents during lactation leads to metabolic disorders, including insulin resistance and increased adipose tissue gene expression of PAI-1 in the adult offspring. These changes could increase the risk for thrombosis [8,9].

In this context, we demonstrated that increased hypothalamic IL-6, TNF-α and IL1-β concentrations contributed to hypothalamic inflammation and impaired satiety-sensing in the offspring with deleterious consequences [10].

On the other hand, several studies in humans and animals have confirmed that dietary fibers such as oligofructose (OF), fructooligosaccharides (FOS) and inulin influence glucose metabolism, specifically by reducing glucose serum concentrations [11-14]. The authors suggested that these dietary fibers affect the digestion and absorption of the carbohydrates and starches [13] resulting in a serum glucose-lowering effect [15,16], improved glucose tolerance and increased insulin sensitivity [13].

Oligofructose, fructooligosaccharides and inulin are members of the inulin-type fructans group. OF is the combination of “non-digestible” oligosaccharides commonly obtained by partial enzymatic hydrolysis of chicory root inulin, linked by ß (2 → 1) linkages of fructosyl units sometimes ending with a glucosyl unit [13,17]. OF and FOS are considered synonyms for inulin-type fructans with a maximum degree of polymerization (DP) of less than 10 [13]. In addition, due to the ß-configuration, OF and other inulin-type fructans are hydrolyzed and fermented by the colonic microbiota rather than being digested in the upper gastrointestinal tract, with health benefits to the host [17-20]. Thus, the inulin-type fructans are considered dietary fibers and prebiotics [17,20,21].

Hyperlipidic diets, especially those rich in saturated fatty acid, increase endotoxemia by the promoting the translocation of lipopolysaccharides (LPS) from the membranes of gram-negative intestinal bacteria [22,23]. LPS-induced TLR-4 activation contributes to systemic inflammation by inducing the secretion of pro-inflammatory cytokines and chemokines. Prebiotics are fermented by the colonic bacteria and can alter the intestinal environment (bacterial population, intestinal permeability), thereby reducing serum LPS concentrations [24].

During pregnancy and lactation, the maternal intake of prebiotics and dietary fibers is considered important and beneficial for the mother and the offspring, from birth through adulthood. In particular, butyrate, the end-product of oligosaccharide fermentation, is a histone deacetylase inhibitor, which is believed to reactivate silent genes by producing epigenetic modifications. These effects on gene activity would be beneficial in the long term [1]. Moreover, a large variety of oligosaccharides are present in human milk at concentrations ranging from 10 to 20 g/L [25,26]. After lactose and fat, oligosaccharides are the most abundant group of human milk components, potentially preventing pathogen adhesion to the intestinal epithelium, influencing the gut maturation process and the intestinal microbiome, and modifying systemic functions such as anti-inflammatory effects [25]. The aim of this study was to evaluate the effect of the supplementation of the diet of the dams with 10% oligofructose in the presence or absence of hydrogenated vegetable fat during pregnancy and lactation on the pro-inflammatory status of 21-day-old pups.

## Materials and methods

### Animals and treatments

The experimental research committee of the Universidade Federal de São Paulo approved all procedures for the care of the animals used in this study followed international recognized guidelines (protocol n°2011/1907). The rats were kept under controlled conditions of light (12-h light/12-h dark cycle with lights on at 07:00) and temperature (24 ± 1°C). Three-month-old female Wistar rats (4 animals in each group) were left overnight to mate, and copulation was verified the following morning by the presence of sperm in vaginal smears.

On the first day of pregnancy, the dams were isolated in individual cages and randomly divided into four groups, each receiving one of four diets: a control diet (C diet, C group), a control diet supplemented with oligofructose (CF diet, CF group), a diet enriched with hydrogenated vegetable fat (T diet, T group) or a diet enriched with hydrogenated vegetable fat supplemented with oligofructose (TF diet, TF group). The diets were maintained throughout pregnancy and lactation.

The four diets were prepared according to the recommendations of the American Institute of Nutrition (AIN- 93G) [27] and were similar in calories and lipid content. The source of lipids for the C and CF diets was soybean oil, and the principal source for the T and TF diets were partially hydrogenated vegetable fat, which is rich in TFAs. The CF and TF diets were prepared by adding 100 g/kg diet of oligofructose (Orafti P95, Pemuco, Chile). According to manufacturer, the OF used in this study is a mixture of oligosaccharides extracted from chicory root. These oligosaccharides are composed of fructose units connected by ß (2–1) links. Some of these molecules are terminated by a glucose unit. The degree of polymerization (DP) of oligofructose in this supplement ranges between 2 and 8.

The centesimal composition of the diets is presented in Table 1. The fatty acid profile of C and T diets was previously described by Pisani et al. [6].

**Table 1:** **Composition of the control diet, control diet supplemented with oligofructose, diet enriched with *trans *fatty acids and diet enriched with *trans *fatty acids supplemented with oligofructose according to AIN-93**

			**Diet (g/100g)**	
Ingredient	C	CF	T	TF
Casein*	20.0	20.0	20.0	20.0
L-cystine†	0.3	0.3	0.3	0.3
Cornstarch†	62.0	52.0	62.0	52.0
Soybean oil‡	8.0	8.0	1.0	1.0
Hydrogenated vegetable fat$	-	-	7.0	7.0
Butylhydroquinone†	0.0014	0.0014	0.0014	0.0014
Mineral mixture£	3.5	3.5	3.5	3.5
Vitamin mixture#	1.0	1.0	1.0	1.0
Cellulose†	5.0	5.0	5.0	5.0
Choline bitartrate†	0.25	0.25	0.25	0.25
Oligofructose¢	-	10.0	-	10.0
Energy (kcal/g)	4.0	4.0	4.0	4.0

*Casein was obtained from Labsynth, São Paulo, Brazil.

†L-cystine, cornstarch, butylhydroquinone, cellulose and choline bitartrate were obtained from Viafarma, São Paulo, Brazil.

‡Oil was supplied from soybean (Lisa/Ind. Brazil).

$Hydrogenated vegetable fat was supplied from Unilever, São Paulo, Brazil.

£Mineral mix 9mg/kg diet): calcium, 5000; phosphorus, 1561; potassium, 3600; sodium, 1019; chloride, 1571; sulfur, 300; magnesium, 507; iron, 35; copper, 6.0; manganese, 10.0; zinc, 30.0; chromium, 1.0; iodine 0.2; selenium, 0.15; fluoride, 1.00; boron, 0.50; molybdenum, 0.15; silicon, 5.0; nickel, 0.5; lithium, 0.1; vanadium, 0.1 (AIN-93G mineral mix, Rhoster, Brasil).

#Vitamin mix (mg/kg diet): thiamin HCL, 6.0, riboflavin, 6.0; pyridoxine HCL 7.0; niacin, 30.0; calcium pantothenate, 16.0; folic acid, 2.0; biotin, 0.2; vitamin B12, 25.0; vitamin A palmitate 4000 IU; vitamin E acetate, 75; vitamin D3, 1000 IU; vitamin KI, 0.75. (AIN-93G, vitamin mix, Rhoster, Brasil).

¢Oligofructose (P95) was manufactured by Orafti (Pemuco, Chile) and was obtained by Viafarma, São Paulo, Brazil.

On the day of delivery, considered day 0 of lactation, litter sizes were adjusted to eight pups each. The pups were weighed and measured (naso-anal length) at birth and on postnatal days 7, 14 and 21.

### Experimental procedures

The pups were decapitated on postnatal day 21. The animals were not fasted to avoid the weaning stress. Trunk blood was collected and immediately centrifuged. The serum was separated and stored at −80°C for later determination of glucose, insulin and adiponectin. The retroperitoneal (RET) white adipose tissue, liver, soleus (SOL) and extensor digital longus (EDL) muscles were isolated, weighed, immediately frozen in liquid nitrogen and stored at −80°C.

### Carcass lipid and protein content

The carcass lipid and protein content was determined in the C, CF, T and TF rat pups. The carcasses were eviscerated, and the RET, epididymal and mesenteric white adipose tissue, SOL, EDL and liver were removed. The remaining carcasses were weighed and stored at −20°C. The lipid content was measured as described by Stansbie et al. [28] and standardized using the method described by Oller do Nascimento and Williamson [29]. Briefly, the eviscerated carcass was autoclaved at 120°C for 90 min and homogenized with water at a volume twice the carcass mass. Triplicate aliquots of this homogenate were weighed and digested in 3 mL of 30% KOH and 3 mL of ethanol for ≥2 h at 70°C in capped tubes. After cooling, 2 mL of 12 N H_2_SO_4_ was added, and the samples were washed three times with petroleum ether to extract the lipids. The results are expressed as grams of lipid per 100 g of carcass. To measure the protein content, aliquots of the same homogenate (approximately 1 g) were heated to 37°C for 1 h in 0.6 N KOH with constant shaking. After clarification by centrifugation, the protein content was measured using the Bradford assay (Bio-Rad, Hercules, California) with bovine serum albumin as a reference.

### Biochemical and hormonal serum analysis

The serum glucose concentrations were measured with a commercial enzymatic colorimetric kit (Labtest, Brazil). The insulin and adiponectin concentrations were quantified using specific enzyme-linked immunosorbent assay (ELISA) kits (Linco Research, USA).

### IL-6, IL-10 and TNF-α protein levels determined by ELISA

Following decapitation, portions of the RET (0.3 g), liver (0.1 g), EDL and SOL (0.1 g) were homogenized in 800 μL of chilled extraction buffer (100 mM Trizma Base pH 7.5; 10 mM EDTA; 100 mM NaF; 10 mM Na_4_P_2_O_7_; 10 mM Na_3_VO_4_; 2 mM PMSF; 0.1 mg/ml aprotinin). After homogenization, 80 μl of 10% Triton X-100 was added to each sample. These samples were held on ice for 30 minutes and then centrifuged (20817 g, 40 minutes, 4°C). The supernatant was saved, and protein concentrations were determined using the Bradford assay (Bio-Rad, Hercules, California) with bovine serum albumin as a reference. Quantitative assessment of TNF-α, IL-6 and IL-10 proteins was carried out using ELISA (DuoSet ELISA, R&D Systems, Minneapolis, MN, USA) following the recommendations of the manufacturer. All samples were run in duplicate, and the mean value is reported.

### Statistical analysis

All results are presented as the means ± standard error of the mean (SE). The statistical significance of the differences between the means of the four groups of samples was assessed using two-way analysis of variance (ANOVA), followed by Tukey's test. Differences were considered to be significant when *p* < 0.05.

## Results

### Body weight, body weight gain, length of the animal and carcass lipid and protein content

**Figure 1:**
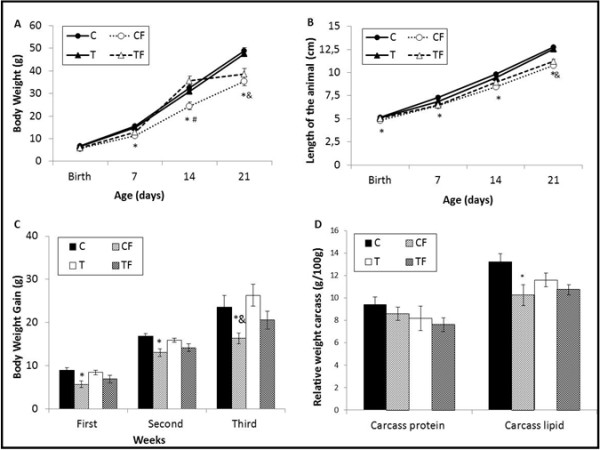
**(A) Body weight, (B) Length, (C) Body weight evolution and (D) Carcass protein and lipid content.** C - mothers fed control diet; CF - mothers fed control diet supplemented with oligofructose; T - mothers fed diet enriched with hydrogenated vegetable fat; TF - mothers fed diet enriched with hydrogenated vegetable fat supplemented with oligofructose. Data are means ± SE of 8–14 determinations per group. ^*^p < 0.05, versus C. ^#^ p < 0.05, versus CF. ^&^ p < 0.05, versus T. ^$^ p < 0.05, versus TF.

The mean body weights (BWs) of the pups at birth were similar among groups. However, the BWs of the 7-d-old pups of the CF group were significantly lower (p = 0.0211) than those of the C group. Similarly, the BWs of the 14-d-old pups of the CF group were lower than those of the C (p = 0.0195) and TF (p = 0.0005) groups. Finally, the BWs of the 21-d-old CF and TF groups were significantly lower than those of the C (p = 0.002; p = 0.0107, respectively) and T (p = 0.0064; p = 0.0309, respectively) groups (Figure 1A).

Regarding the BW gain, the CF group showed lower gains in the first (p = 0.0233) and second (p = 0.0217) weeks, compared to the C group. In the third week, the BW gain in the CF group was significantly lower than the C (p = 0.0112) and T (p = 0.0004) groups (Figure 1C).

Figure 1B shows that lengths of the pups in the CF group were significantly lower at birth (p = 0.0315) and at 14-d-old (p = 0.0198) than those of the C group at the same time points. The lengths of the 21-d-old pups of the CF and TF groups were lower than those of the C (p < 0.0001; p = 0.0002, respectively) and T (p = 0.0003; p = 0.0022, respectively) groups.

The relative carcass lipid content in the CF group was significantly lower than the C (p = 0.0422) group. However, the relative carcass protein content was similar among the C, CF, T and TF groups (Figure 1D).

### Relative weight of tissues

The relative RET weight in the CF group was significantly lower than that of the C (p < 0.0001) and T (p < 0.0001) groups. There were no differences in relative liver weight among groups. The relative SOL weight in the CF group was lower than that of T (p = 0.0166) group. The relative EDL weights of the CF and TF groups were lower than those of the C (p = 0.0057; p = 0.0132) and T (p = 0.0021; p = 0.0051) groups (Table 2).

**Table 2:** Relative weight of tissues adipose retroperitoneal (RET), soleus (SOL) and long extensor digit (EDL) muscles and liver in pups with 21 days

**Relative weight (g/100g b.w.)**	**C (9)**	**CF (10)**	**T (9)**	**TF (9)**
RET	0.45 ± 0.04	0.27 ± 0.04^*,&^	0.45 ± 0.04	0.33 ± 0.02
Liver	3.31 ± 0.06	3.13 ± 0.08	3.27 ± 0.06	3.44 ± 0.08
SOL	0.07 ± 0.00	0.06 ± 0.00^&^	0.07 ± 0.00	0.06 ± 0.00
EDL	0.07 ± 0.00	0.06 ± 0.00^*,&^	0.07 ± 0.00	0.06 ± 0.00^*,&^

C - mothers fed control diet; CF - mothers fed control diet supplemented with oligofructose; T - mothers fed diet enriched with hydrogenated vegetable fat; TF - mothers fed diet enriched with hydrogenated vegetable fat supplemented with oligofructose. The number in parentheses refers to the sample value. Data are means ± SE. ^*^p < 0.05, versus C. ^&^ p < 0.05, versus T.

### Serum glucose, adiponectin and insulin concentrations

The serum concentrations of glucose and insulin of the pups at 21-d-old were similar among all groups. However, the serum levels of adiponectin were significantly lower in the CF (p < 0.0001), T (p = 0.0013) and TF (p = 0.0038) groups compared with the C group (Table 3).

**Table 3:** Serum glucose, adiponectin and insulin in pups with 21 days

	**C**	**CF**	**T**	**TF**
Glucose (mg/dL)	107.95 ± 3.43 (9)	116.03 ± 5.38 (10)	112.07 ± 3.92 (9)	119.82 ± 3.22 (9)
Adiponectin (μg/mL)	24.13 ± 3.64 (16)	14.12 ± 1.52^*^ (16)	15.89 ± 2.02^*^ (16)	16.59 ± 2.36^*^ (16)
Insulin (ng/mL)	0.23 ± 0.02 (9)	0.27 ± 0.03 (10)	0.30 ± 0.04 (10)	0.35 ± 0.03 (9)

C - mothers fed control diet; CF - mothers fed control diet supplemented with oligofructose; T - mothers fed diet enriched with hydrogenated vegetable fat; TF - mothers fed diet enriched with hydrogenated vegetable fat supplemented with oligofructose. The number in parentheses refers to the sample value. Data are means ± SE. ^*^p < 0.05, versus C.

### Tissues cytokine content

**Figure 2:**
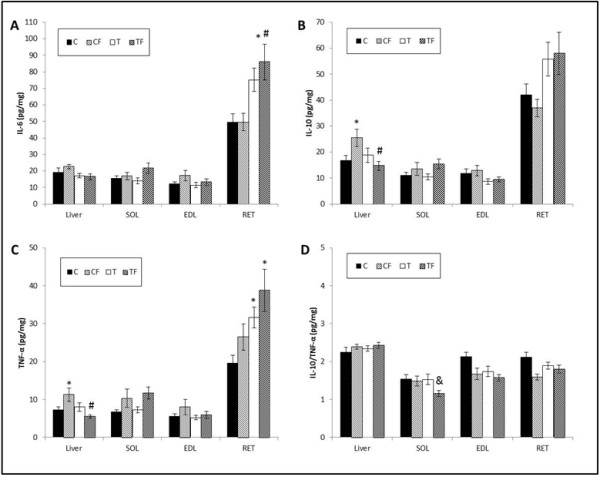
**(A) IL-6 content in adipose retroperitoneal (RET), soleus (SOL), long extensor digit (EDL) muscle and liver, (B) IL-10 content in RET, SOL, EDL and liver, (C) TNF-α content in RET, SOL, EDL and liver and (D) IL-10/TNF-α ratio in RET, SOL, EDL and liver.** C - mothers fed control diet; CF - mothers fed control diet supplemented with oligofructose; T - mothers fed diet enriched with hydrogenated vegetable fat; TF - mothers fed diet enriched with hydrogenated vegetable fat supplemented with oligofructose. Data are means ± SE of 6–17 determinations per group. ^*^p < 0.05, versus C. ^#^ p < 0.05 versus CF. ^&^ p < 0.05, versus T. ^$^ p < 0.05 versus TF.

Figure 2A shows that the IL-6 content in the RET was greater in the TF group than in the C (p = 0.0203) and CF (p = 0.0106) groups. In other tissues, the IL-6 content was not significantly different among the C, CF, T and TF groups.

The IL-10 content in the liver was greater in the CF group than in the C (p = 0.0041) and TF (p = 0.0021) groups. In RET, SOL and EDL, the IL-10 content did not differ among the groups (Figure 2B).

The TNF-α content of the livers of the CF group was greater than that of the C (p = 0.0027) and TF (p = 0.0005) groups. In RET, the TNF-α content in the T (p = 0.0462) and TF (p = 0.0037) groups was greater than that of the C group (Figure 2C).

The IL-10/TNF-α ratio was lower in SOL of the TF group compared to the T (p = 0.0073) group (Figure 2D), but not in any other tissues or groups.

## Discussion

In the present study, supplementation of the dams diet with 10% oligofructose during pregnancy and lactation reduced the body weight, body weight gain, length and lipid carcass content; increased levels of TNF-α and IL-6; and decreased the serum levels of adiponectin of the pups. These results indicate that supplementation of the dam’s diet with a high oligofructose content (10%) induced a pro-inflammatory state in the offspring.

The 21-d-old pups of the CF and TF groups had lower body weights and lengths compared to those of the C and T groups. The body weight and length effects were accompanied by a reduction in the relative weights of the RET, SOL and EDL. Decreased body weight gain was also observed in the pups of the CF group throughout the experimental period.

Previous studies regarding the effect of high fiber diets during pregnancy and lactation indicate controversy in the field. In agreement with our results, Carabin and Flamm reported that the ingestion of diet containing 20% FOS during pregnancy and lactation caused a delay in the growth of the pups [13].

In contrast, other studies did not report negative effects of FOS supplementation during pregnancy on the development of offspring [13,30]. Furthermore, Pisani et al. previously reported that trans fatty acid intake during pregnancy and lactation did not modify the body weight of the pups during the entire period of lactation. In our study, the birth weights were not affected either by a trans fatty acid diet or 10% OF supplementation during pregnancy [6]. Maurer et al. showed no differences in the birth weight or subsequent body weights of the pups at 7, 14, or 21 days after birth from dams fed with control, high fiber (a combination of the prebiotic fibers inulin and oligofructose) or high protein diet during pregnancy and lactation. Similarly, Rodenburg et al. reported no difference in body weight gain of eight-week-old rats fed a control diet or one containing 6% of FOS [31,32].

In addition, the change in weight gain and reduced carcass lipid content found in the CF group compared to the C group is consistent with the hypothesis that offspring of dams fed a diet supplemented with high amount of oligofructose suffer from malnutrition. Taken together, these results suggest that the amount and type of prebiotic ingested as well as the period during which the animal is exposed could influence the development of the animal.

Trans fatty acid intake during pregnancy and lactation increased the TNF-α content in the pup’s RET. Previously, it has been shown that ingestion of hydrogenated vegetable fat rich in TFAs during these periods decreased AdipoR1 and elevated TRAF-6 protein expression in the RET of 21-d-old offspring. PAI-1 and TNF-α gene expression was also increased, while the serum adiponectin levels decreased [7,9]. Furthermore, TNF-α gene expression in adipose tissue has been shown to correlate with circulating TNF-α levels [33].

The oligofructose supplementation did not alter the inflammatory effect of the trans fatty acids. In fact, the analysis of the IL-6 content in RET suggested that the addition of OF seemed to increase the inflammatory effect of trans fatty acids. On the other hand, the supplementation of control diet with OF (10%) during pregnancy and lactation caused an increase in TNF-α and IL-10 in the 21-d-old pups’ livers. This pro-inflammatory environment was accompanied by a reduction in the plasma adiponectin concentrations in CF, T and TF. Adiponectin increases insulin sensitivity and has anti-inflammatory and anti-atherogenic effects [34]. In the literature, increased TNF-α concentrations were reported to be associated with a decrease in the expression and secretion of adiponectin [35,36].

In a review, Roberfroid reported several studies that identified health benefits of FOS in animals and humans and addressed possible intolerance [21]. These studies demonstrated that the symptoms are dose-dependent: specifically, diarrhea can develop with intakes of 30 g/d or more in humans.

In our study, the supplementation of diet with 10% OF during pregnancy and lactation caused diarrhea in dams (data not shown). This effect could have contributed to development of pro-inflammatory environment observed in the offspring. Bruggencate et al. studied supplementation with 3% or 6% of FOS and found that the supplement impaired resistance to intestinal infections, with dose-dependent increases in the colonization and translocation of Salmonella enteritidis [37]. In addition, FOS decreased cecal and fecal pH in a dose-dependent manner. Altered pH in these compartments can contribute to impairment of the intestinal mucosal barrier through colonic mucosal damage and/or inflammation associated with the rapid production of the short chain fatty acids (SCFA) and/or lactate (organic acids). Moreover, Rodenburg et al. reported that the intake of FOS by rats harmed the barrier integrity by increasing mucosal permeability, bacterial translocation and expression of colonic mitochondrial genes that may be involved in the maintenance of the intestinal barrier [32]. FOS stimulates the growth, not only of potentially beneficial bacterial species including bifidobacteria and enterobacteria but also of potential pathogens [37]. It is also possible that the oligosaccharides presents in maternal milk [25] can lead to excessive production of organic acids in the gut of pups, altering the intestinal permeability and microbiome composition in the offspring [32,37]. Thus, an alteration of bacterial population and an increase in bacterial translocation and intestinal permeability could cause an increase in serum LPS, resulting in TLR4-mediated inflammatory responses [24].

## Conclusion

In conclusion, supplementation of the diet of the dams with oligofructose (10%) during pregnancy and lactation, in the presence or absence of supplementation with hydrogenated vegetable fat, adversely affected the development of the offspring and contributed to increases in the pro-inflammatory status in pups at postnatal day 21. Further studies are needed in this area to examine the dose-dependent effects of the oligofructose on metabolic programming.

## Competing interests

The authors declare that they have no competing interests.

## Authors’ contributions

ACLH - designed the study, carried out the experiments, performed the statistical analysis and drafted the manuscript. LVM - designed the study, carried out the experiments, performed the statistical analysis and drafted the manuscript. JLO - participated in the design of the study helped to carried out the experiments. MFM - helped to carried out the experiments. MHO - helped to carried out the experiments. BS - helped to carried out the experiments. LMO- helped to carried out the experiments, revised and helped to draft the manuscript. EBR - helped to draft the manuscript. CMON- conceive the study, participated in its design, and helped to draft the manuscript. LPP - conceive the study, participated in its design, and helped to draft the manuscript. All authors read and approved the final manuscript.
